# Transcriptome signature of cell viability predicts drug response and drug interaction in *Mycobacterium tuberculosis*

**DOI:** 10.1016/j.crmeth.2021.100123

**Published:** 2021-12-06

**Authors:** Vivek Srinivas, Rene A. Ruiz, Min Pan, Selva Rupa Christinal Immanuel, Eliza J.R. Peterson, Nitin S. Baliga

**Affiliations:** 1 Institute for Systems Biology, Seattle, WA, USA; 2 Departments of Biology and Microbiology, University of Washington, Seattle, WA, USA; 3 Molecular and Cellular Biology Program, University of Washington, Seattle, WA, USA; 4 Lawrence Berkeley National Lab, Berkeley, CA, USA

**Keywords:** tuberculosis, drug-resistant tuberculosis, TB drug discovery, drug response prediction, drug combinations, drug synergy, TB treatment regimens, computational model, machine learning

## Abstract

There is an urgent need for new drug regimens to rapidly cure tuberculosis. Here, we report the development of drug response assayer (DRonA) and “MLSynergy,” algorithms to perform rapid drug response assays and predict response of *Mycobacterium tuberculosis* (Mtb) to drug combinations. Using a transcriptome signature for cell viability, DRonA detects Mtb killing by diverse mechanisms in broth culture, macrophage infection, and patient sputum, providing an efficient and more sensitive alternative to time- and resource-intensive bacteriologic assays. Further, MLSynergy builds on DRonA to predict synergistic and antagonistic multidrug combinations using transcriptomes of Mtb treated with single drugs. Together, DRonA and MLSynergy represent a generalizable framework for rapid monitoring of drug effects in host-relevant contexts and accelerate the discovery of efficacious high-order drug combinations.

## Highlights

• DRonA defines the transcriptome signature for *M. tuberculosis* (Mtb) viability
• DRonA detects killing of Mtb by diverse mechanisms and in diverse contexts
• DRonA predicts viability of dormant non-culturable Mtb cells
• DRonA with MLSynergy predicts the outcome of two- and three-drug combinations

## Motivation

The treatment of tuberculosis (TB), which kills 1.8 million each year, remains difficult, especially with the emergence of multidrug resistant strains of *Mycobacterium tuberculosis* (Mtb). While there is an urgent need for new drug regimens to treat TB, the process of drug evaluation is slow and inefficient, owing to the slow growth rate of the pathogen, the complexity of performing bacteriologic assays in a high-containment facility, and the context-dependent variability in drug sensitivity of the pathogen. The algorithms “DRonA” and “MLSynergy” described here use transcriptomes of drug-treated Mtb to predict drug response and drug interaction in diverse contexts.

## Graphical abstract

**Figure:**
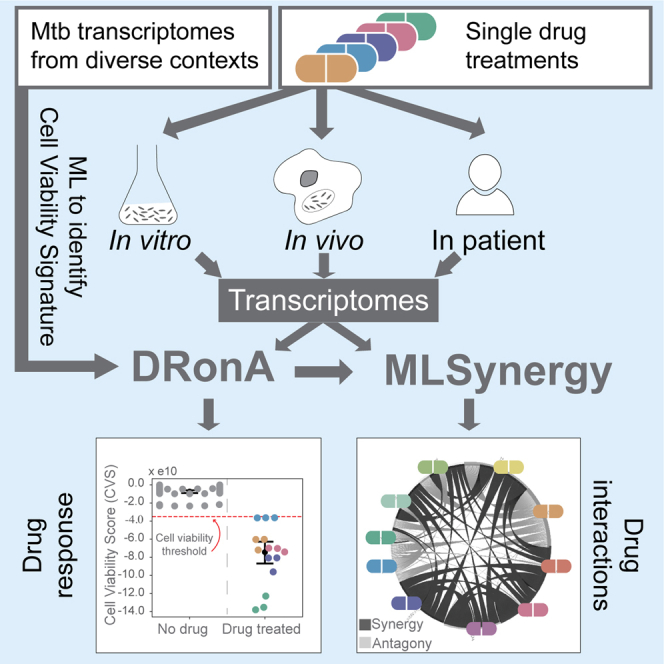

## Teaser

To develop new, shorter tuberculosis drug regimens, there is a critical need to easily and rapidly measure treatment efficacy. Srinivas et al. report the development of DRonA and MLSynergy algorithms, which leverage transcriptomic signatures of viability for *in silico* prediction of Mtb drug response and multidrug interactions.

## Introduction

New treatment regimens containing multiple drugs are needed to achieve rapid and complete clearance of *Mycobacterium tuberculosis* (Mtb), the causative agent of tuberculosis (TB). However, the discovery of effective multidrug combinations is a challenging endeavor, burdened by the enormous number of testable combinations (e.g., a collection of 1,000 compounds yields ∼500,000 pairwise combinations and exponentially larger numbers of higher-order combinations). Multicomponent drug discovery is particularly challenging for Mtb, a slow-growing pathogen that is capable of generating phenotypically heterogeneous subpopulations. These phenotypically diverse subpopulations allow Mtb to persist and survive the variable conditions encountered during infection as well as thwart drug treatment. Because of drug-tolerant subpopulations within the host, a large proportion of drug regimens that are effective in killing Mtb *in vitro* are futile in patients (Ji et al., 1996; Sarathy et al., 2018, 2019). Suffice it to say, new approaches are needed to reduce the search space and prioritize combinations for experimental testing, while also taking into account the host context and different subpopulations of Mtb.

Another challenge in the development of new antitubercular drug regimens is the reliance on growth assays to monitor treatment response. Current methods to monitor treatment response include counting of colony forming units (CFUs) on solid agar plates and measuring the time it takes for a sample in liquid culture to become culture positive for Mtb, in what is termed time to positivity (TTP) assay. Both CFU counting and TTP have their drawbacks including loss of sensitivity, vulnerability to contamination, and lengthy time to measure results. Furthermore, culture on solid media or in liquid media requires actual growth, which limits the detection of mycobacterial subpopulations that are viable but not actively growing (Diacon et al., 2012). Instead, profiling 16S ribosomal RNA as a proxy for Mtb load in sputum is emerging as a more sensitive technique that addresses the shortcomings of growth-based assays (Honeyborne et al., 2011, 2014). This is a promising development because information in RNA can be amplified using technologies such as probe capture and PCR to develop highly sensitive methods for investigating the drug response of Mtb, especially from patient samples. Furthermore, genome-wide expression studies of Mtb from broth, sputum, and *in vivo* infections have been used to uncover physiologic states and transcriptional mechanisms of drug tolerance (Honeyborne et al., 2016; Peterson et al., 2019). Here, we have investigated if transcriptome profiling of Mtb can report on the effect of drug treatment, and whether this information can also enable *in silico* identification of drug combinations that are likely to have synergistic or antagonistic effects on the pathogen.

We report the development of a framework of two algorithms drug response assayer (DRonA) and “MLSynergy” that can use transcriptomes to predict Mtb’s response to drug treatment and classify two- and three-drug combinations based on the likelihood of synergistic or antagonistic action on Mtb. DRonA is a machine learning algorithm that was trained on publicly available transcriptomes of Mtb cultured in diverse conditions (with and without perturbation) to detect a gene signature for loss of Mtb viability. Using drug-induced transcriptional changes, DRonA calculates a cell viability score (CVS), which distinguishes the extent of a drug’s bacteriostatic or bactericidal activity on Mtb. We demonstrate that DRonA accurately detects within the transcriptome profile of drug-treated Mtb evidence for loss of bacterial viability, regardless of the drug’s mechanism of killing. Furthermore, DRonA was equally accurate in determining drug-induced viability reduction in Mtb from broth culture, macrophage infection, and patient sputum. Finally, MLSynergy uses transcriptomes from single-drug treatment to predict the interaction of drugs in combination. Using the ratio of the expected CVS (based on CVSs of individual drugs) and the predicted CVS (based on the inferred multidrug transcriptome) calculated by DRonA, MLSynergy can distinguish between synergistic and antagonistic combinations. We demonstrate that MLSynergy accurately classified experimentally determined synergistic and antagonistic combinations. Thus, the DRonA/MLSynergy framework can accelerate antitubercular drug discovery by reducing the reliance on growth-based treatment response assays and guiding the experimental assessment of novel drug combinations.

## Results

### DRonA detects signatures for loss of viability within transcriptomes of Mtb, irrespective of mechanism of killing

**Figure 1:**
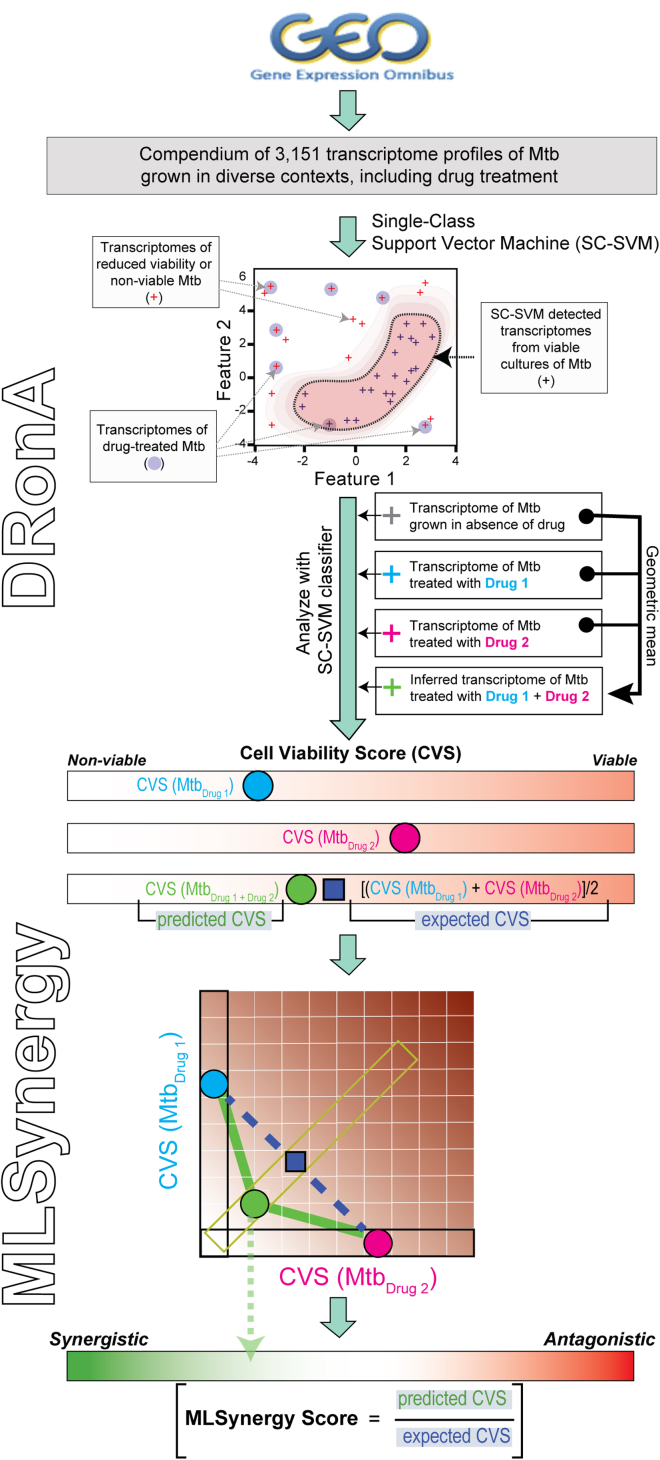
Overview schematic of DRonA/MLSynergy framework DRonA is a SC-SVM that was trained on transcriptomes from viable Mtb cultures. DRonA was trained through an iterative process to define a region in the hyperplane that classifies transcriptomes from Mtb grown in varying growth conditions as viable and distinguishes them from non-viable transcriptomes (i.e., drug treated at >MIC_50_ concentration). DRonA takes transcriptomes as input and outputs a CVS, which is the empirical distance from the viable class and indicative of efficacious drug treatment. Using an inferred transcriptome of a drug combination from single-drug transcriptomes, MLSynergy predicts the outcome of the drug interaction. MLSynergy uses the Loewe additivity principle and calculates the ratio of predicted CVS to expected CVS to determine synergy or antagonism for drug combinations.

To investigate whether Mtb viability can be deciphered from its transcriptome state, we sought to define a classifier that could accurately identify transcriptomes of viable Mtb. We hypothesized that the degree of deviation of a transcriptome from the boundary defined by the above-mentioned classifier would indicate the loss of viability of Mtb cells. Further, we hypothesized that the loss of viability would be agnostic of the inhibitory effect, making it possible to predict drug-mediated killing, irrespective of the mechanism of action (Figure 1). While there are many good classification techniques (e.g., artificial neural networks, decision trees, Bayesian classifiers), the support vector machine (SVM) algorithm is one of the best techniques to optimize the expected solution (i.e., identifying a signature of viable states of Mtb) with limited datasets. Moreover, classification based on SVM offers potential for feature analysis to identify specific genes whose expression levels are diagnostic of the viability state of Mtb. Therefore, we trained a single-class support vector machine (SC-SVM) using a compendium of 3,151 transcriptomes of Mtb grown in diverse conditions to accurately identify the transcriptomes that belong to “viable” states of Mtb. The compendium of 3,151 transcriptomes was compiled from 173 studies available in the Gene Expression Omnibus (GEO). These studies used microarray and RNA sequencing (RNA-seq) to assess gene expression changes in Mtb from various growth medium compositions, culture conditions, and drug treatment (Table S1). Batch effects and platform-specific bias across the transcriptome profiles were corrected with rank normalization, and each profile was labeled as “viable,” “non-viable,” or “unclassified” by manual inspection of the associated metadata. Specifically, 24 transcriptomes of Mtb cultured in optimal growth conditions (mid-log phase of growth in 7H9 nutrient-rich media, incubated at 37°C with aeration) were labeled as “viable,” and 193 transcriptomes of Mtb cultures treated with 17 different drugs at >1× MIC_50_ for >12 h were labeled as “non-viable”. The remaining 2,319 transcriptomes were labeled as “unclassified.” The labeled transcriptome compendium was used for SC-SVM training, which was performed to broaden the classifier-defined boundary of viability by iteratively including transcriptomes from the “unclassified” set that are from viable Mtb adapted to non-lethal, sub-optimal growth conditions (see STAR Methods for details). The classifier was iteratively trained on the “viable” set until addition of transcriptomes from the “unclassified” set caused a drop in its performance in accurately classifying viable and non-viable transcriptomes (Figure S1). The final classifier was trained on 994 transcriptomes (Table S1) of Mtb from diverse growth conditions, including log phase, vehicular control samples, nutrient starvation, low pH, hypoxia, and intracellular growth. As such, the SC-SVM classifier identified Mtb transcriptomes from slow-growing (i.e., dormancy-inducing), but viable conditions. In contrast, the excluded transcriptomes (total 1,940) (Table S1) were from stressful conditions (e.g., drug treated, heat treated, amino acid starved) and lethal genetic perturbations (e.g., *phoP*, *espR*, *mihF* mutants) that reduced cell viability in Mtb cultures (Foddai et al., 2010; Odermatt et al., 2018; Pérez et al., 2001; Roy et al., 2020; Tiwari et al., 2018).

**Figure 2:**
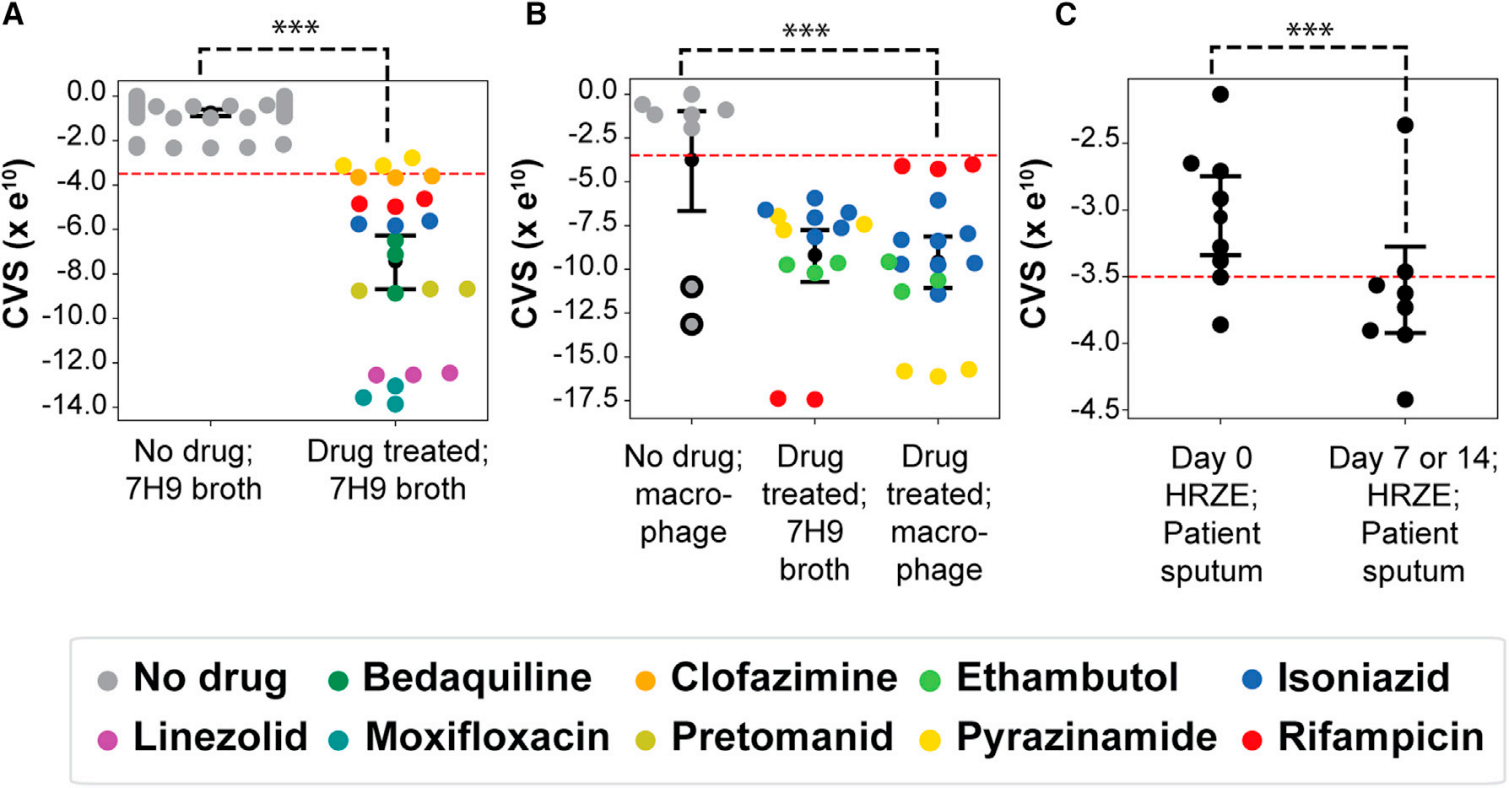
DRonA-generated CVSs for transcriptomes of Mtb sourced from broth culture, macrophage infection, and patient sputum (A) CVSs for transcriptomes of Mtb cultures grown in 7H9-rich media with or without drug treatment for 72 h. (B) CVSs for transcriptomes of Mtb cultured in 7H9 broth with drug treatment for 24 h and macrophage with or without drug treatment for 24 h. Circles with black border indicate transcriptomes from interferon-gamma-activated macrophages with lipopolysaccharide treatment. (C) CVSs for transcriptomes of Mtb in patient sputum collected at the start and end of 7 or 14 day chemotherapy with HRZE: isoniazid (H), rifampicin (R), pyrazinamide (Z), and ethambutol (E). Red dashed line is the cell viability threshold (−3.5e^10^), below which the samples are considered to be non-viable. Black dot and error bars indicate the mean and standard deviation away from the mean. Statistical significance (black dashed line) was calculated as p value with Student’s t test. ∗∗∗: p value < 0.001.

The linear SC-SVM classifier, named **d**rug **r**esp**on**se **a**ssayer (DRonA), can take as input transcriptomes of Mtb to calculate a CVS (see STAR Methods for details). The calculated CVS is proportional to the deviation of a given transcriptome from the lower limit of the classifier-defined viable transcriptome space. This lower limit is set as the cell viability threshold (cell viability threshold = −3.5e^10^), below which a CVS indicates a transcriptome signature of nonviable Mtb. Using an independent compendium of 72 transcriptomes generated for this study (Table S2), we ascertained that the CVS scoring scheme of DRonA accurately classified as “viable” (i.e., with a CVS > −3.5e^10^) all 27 transcriptomes of Mtb grown in 7H9 medium in the absence of drugs. By contrast, DRonA predicted loss of viability (CVS < −3.5e^10^) from transcriptomes of Mtb cultures treated for 72 h in 7H9 growth medium with each of the seven frontline TB drugs at ≥ MIC_50_ concentration (p value < 0.001, Figure 2A). As expected, pyrazinamide treatment at 3.0 mg/mL was not predicted to reduce the viability of Mtb (Peterson et al., 2015). Next, we tested the performance of DRonA in predicting Mtb viability within an intracellular host context, using as input 39 transcriptomes of Mtb from naive, lipopolysaccharide (LPS)-activated, and drug-treated infected macrophages of J774A.1 lineage (Table S2) (Liu et al., 2016). Again, DRonA correctly classified the transcriptomes from untreated Mtb as viable and the drug-treated transcriptomes as non-viable (Figure 2B). Moreover, DRonA detected the known increase in the intracellular efficacy of pyrazinamide (Salfinger et al., 1990; Zhang et al., 2002) and also the decreased efficacy of rifampicin (Adams et al., 2011) in killing Mtb within macrophages. DRonA also detected a loss in the viability of Mtb within interferon-gamma-activated macrophages upon LPS treatment (Fang et al., 2020). Together this demonstrates that DRonA was able to identify non-viable transcriptomes, irrespective of the context and underlying mechanism of killing (i.e., whether immune or drug induced). Finally, we tested the performance of DRonA in predicting drug response within TB patients, using as input 16 transcriptomes of Mtb from the sputum of eight patients at the start of and after 7 or 14 days of successful TB treatment with isoniazid (H), rifampicin (R), pyrazinamide (Z), and ethambutol (E) (Honeyborne et al., 2016). DRonA efficiently differentiated cell viability from the Mtb transcriptomes collected from patients on day 0 from transcriptomes collected on day 7 or 14 of drug treatment (p value < 0.01) (Figure 2C), demonstrating that DRonA can detect drug treatment response from bacterial RNA in patient sputum.

### DRonA can estimate the decline in CFUs upon drug treatment

**Figure 3:**
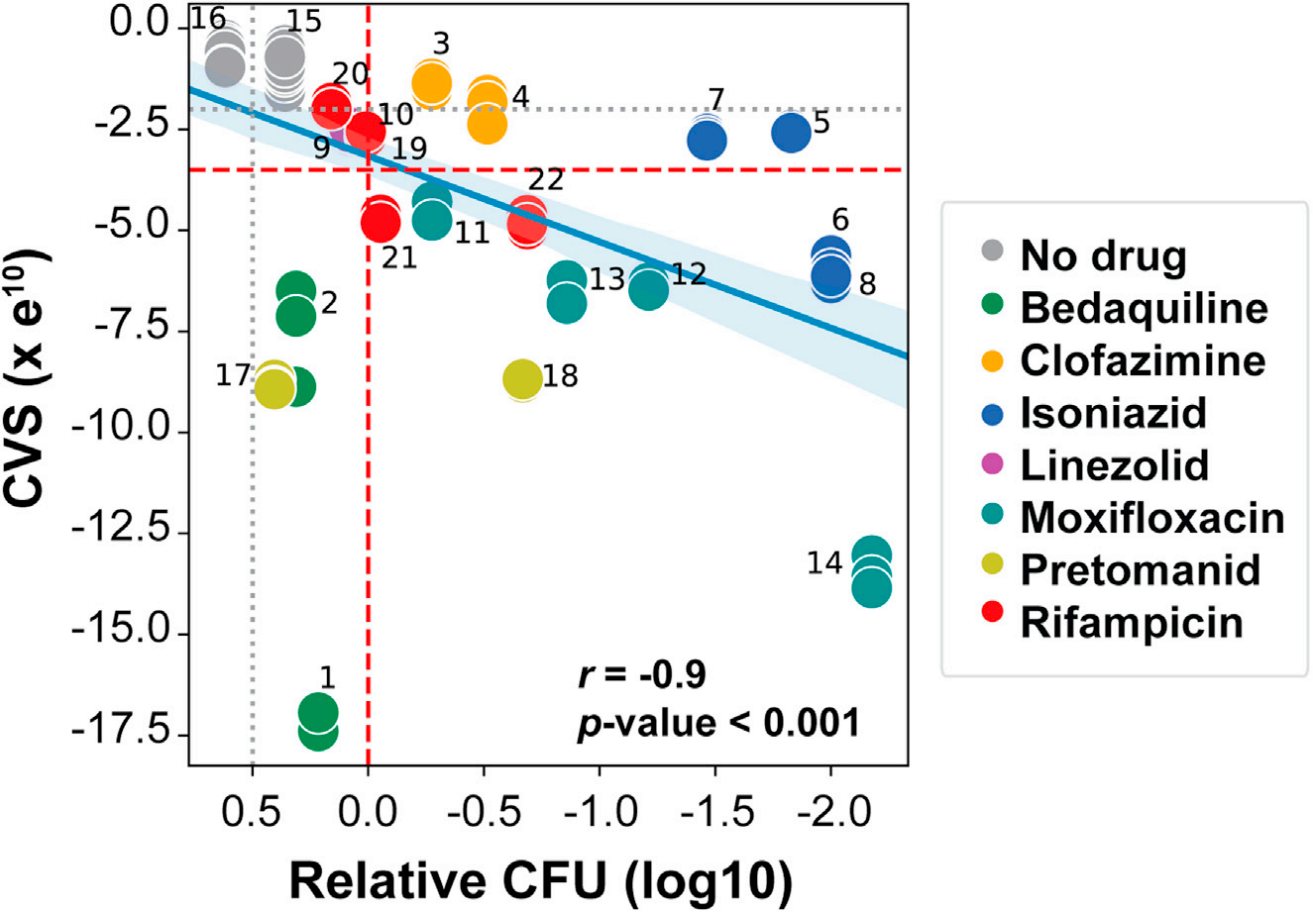
Correlation between CVS and relative CFU with and without drug treatment Relative CFU was calculated in relation to 0 h (prior to drug or vehicle control treatment). Numbers associated with the points indicate specific drug treatment time and concentrations found in Table S3. Relative CFUs for the treatments in Table S3 were calculated with time-kill assay and are given in Table S4. Solid blue line denotes the Pearson’s correlation between CVS and relative CFU. Significance was calculated as the average correlation coefficient, *r*, from 100 iterations performed with 70% randomly selected data. Shaded blue region denotes the variation in *r* from 100 iterations. Black dotted line denotes 50% growth inhibition from drug treatments and its corresponding CVS threshold (−2.25e^10^). Dashed red line indicates bactericidal activity and its corresponding CVS threshold (−3.5e^10^).

We next tested whether the CVS was proportional to the magnitude of drug effects based on CFU assessment. We compared DRonA-generated CVSs with the relative CFUs observed after Mtb was treated for 24 and 72 h with seven frontline TB drugs at various concentrations and conditions (Tables S3 and S4). The CVS scores calculated from transcriptomes of both untreated Mtb cultures and those treated with drugs at < MIC_50_ concentrations were higher than the viability threshold. Although, the inferred CVS from cultures treated with <MIC_50_ drug was less than the CVS of untreated cultures (difference in average = −3.5 × e^10^, p value < 0.01), indicating a moderate loss of viability. In contrast, the CVS scores calculated from transcriptomes of Mtb cultures treated with ≥MIC_50_ concentration of drug were consistently below the viability threshold. Furthermore, for both Mtb grown in 7H9 medium and within macrophages, the reduction in CVS was proportional to the decrease in CFU for most drugs (Figures 3 and S2), with the exception of bedaquiline. It is known that bedaquiline kills Mtb relatively slowly compared with other frontline drugs and could explain the discord between CFU and CVS within 72 h of treatment (Andries et al., 2005; Koul et al., 2007, 2008, 2014; Peterson et al., 2016). It is remarkable that despite the slow bactericidal activity of bedaquiline, its lethal effect was captured in transcriptome changes at a significantly earlier time point, and we still observed a significant correlation between relative CFUs and CVS across all drug treatments (*r* = −0.9, p value < 0.001).

**Figure 4:**
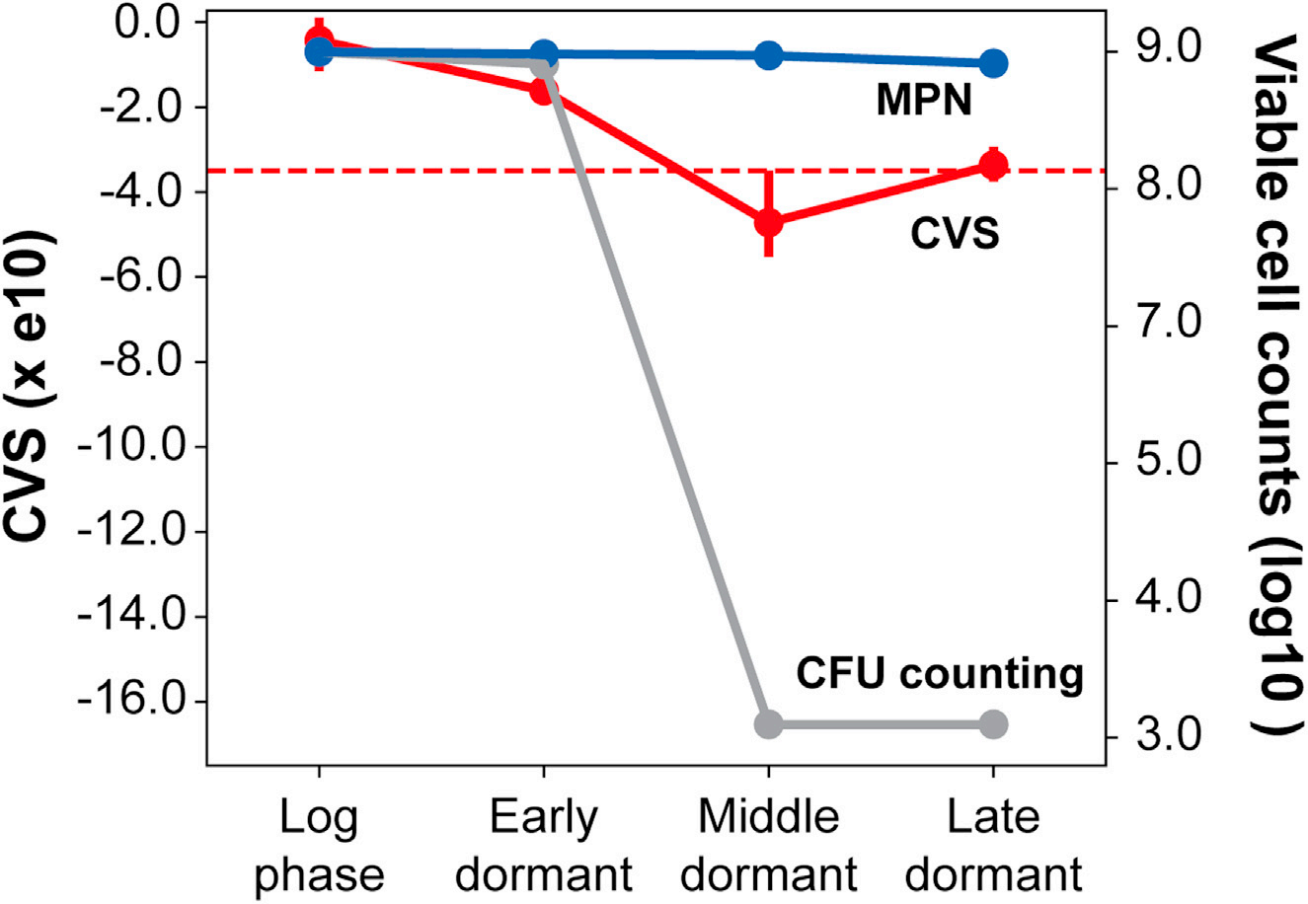
Comparison of CVS from DRonA with bacteriological assays determined by most probable number (MPN) assay and colony forming unit (CFU) enumeration from heterogeneous K+ starved cultures of Mtb Transcriptomes for DRonA prediction were obtained from GEO accession number GSE66408 and viable cell counts according to MPN and CFU counting assays were obtained from Ignatov et al. (2015). Log phase is the exponentially growing cultures of Mtb collected prior to K+ starvation, and early, middle, and late dormant are the rifampicin (5 μg/mL)-treated cultures collected after 10, 20, and 30 days of K+ starvation. Red, blue and grey dots indicate mean, and error bars indicate standard deviation from the mean. The dashed red line is the CVS threshold (−3.5e^10^) from DRonA and indicates loss of cell viability.

A notable disadvantage of performing drug response assessment via CFU counting is the limitation that it only measures culturable bacteria (Dusthackeer et al., 2019). Mtb from *in vivo* models of latent TB infection are non-culturable and require resuscitation-promoting factors or conditions to resume growth (Biketov et al., 2007; Dhillon et al., 2004). Thus, CVS scores determined using mRNA signatures represent a comprehensive assay of drug effects on dormant Mtb that are unable to grow on solid medium but retain full potential of recovering to a physiologically active state. To test this hypothesis, we investigated the accuracy of DRonA in predicting Mtb killing by a moderate concentration of rifampicin (5 μg/mL) in potassium-deficient growth medium (Ignatov et al., 2015). Mtb shifts to a dormant state that is unable to grow on solid medium, but able to recover and proliferate in albumin, dextrose, and sodium chloride (ADC)-supplemented Sauton medium containing potassium (Salina et al., 2019). The results demonstrated that CFU counting overestimated rifampicin-treatment-induced killing of the pathogen, as demonstrated by a minimum probable number (MPN) performed in the same context in ADC-supplemented liquid Sauton medium. Notably, similar to MPN results, there was no significant drop in CVS, demonstrating that DRonA accurately predicted the overall drug response in cultures that consist of non-culturable Mtb (Figure 4).

### MLSynergy accurately predicts synergistic and antagonistic drug combinations from transcriptomes of single-drug-treated Mtb

**Figure 5:**
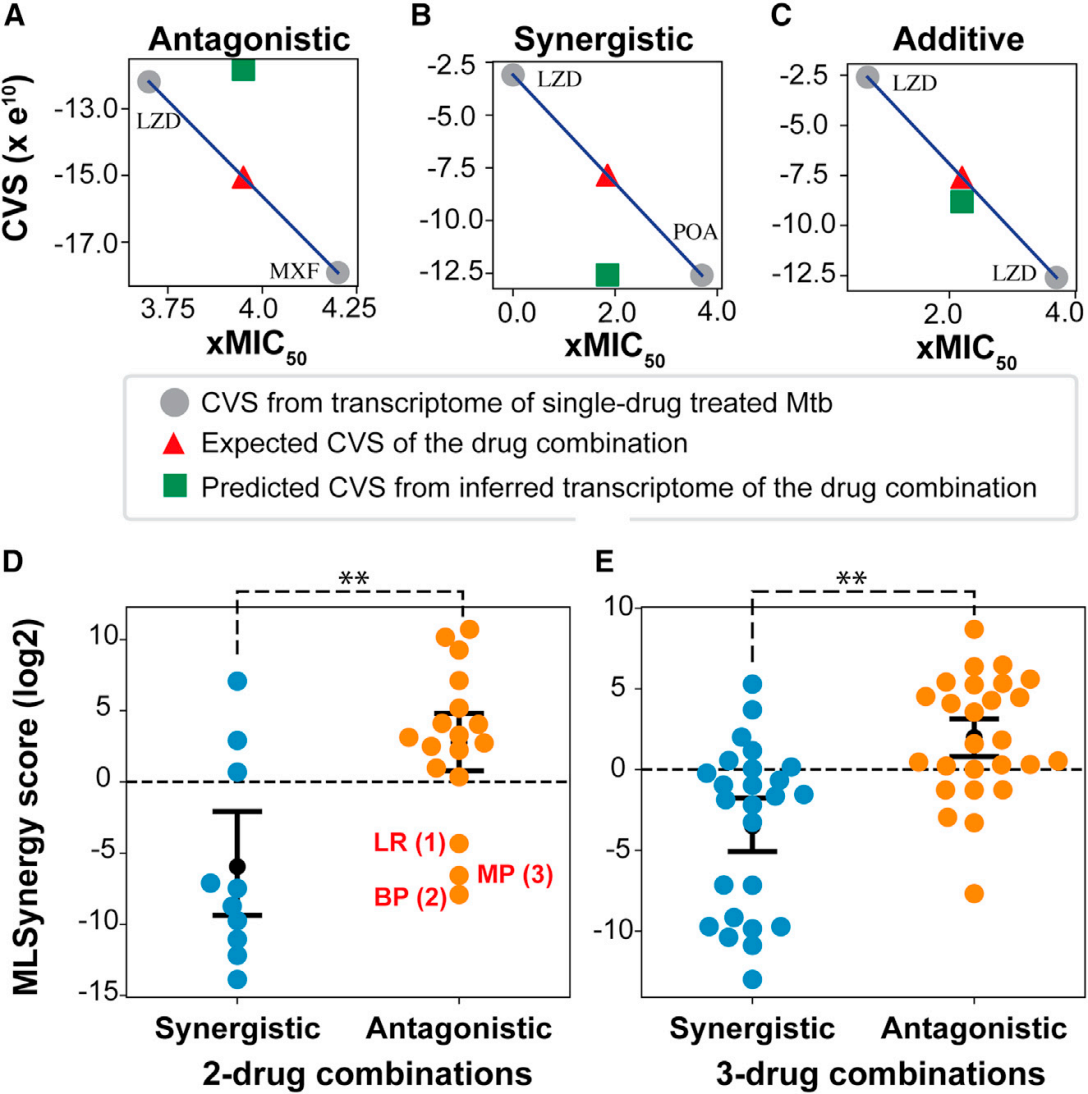
MLSynergy prediction of drug interaction (A–C) Examples of the relationship between expected CVS and predicted CVS for (A) antagonistic, (B) synergistic, and (C) additive drug combinations. The expected CVS (red triangle) was calculated as the average of DRonA-generated CVSs for experimentally measured transcriptomes from single-drug-treated Mtb. LZD; linezolid, POA; pyrazinoic acid, MXF; moxifloxacin. (D) MLSynergy classification of experimentally validated synergistic and antagonistic two-drug combinations. Drug combinations labeled in red: (1) LR; linezolid and rifampicin (Maltempe et al., 2017), (2) BP; bedaquiline and pretomanid (Cokol et al., 2017), and (3) MP; moxifloxacin and pretomanid (de Miranda Silva et al., 2019) were classified as synergistic by MLSynergy and experiments from literature, but antagonistic by Larkins-Ford et al. (2021). (E) MLSynergy classification of experimentally validated synergistic and antagonistic three-drug combinations. Black dot and error bars indicate the mean and standard deviation away from the mean. Statistical significance (black dashed line) was calculated as p value with Student’s T test. ∗∗: p value < 0.01.

Given that DRonA can detect Mtb’s response to drug treatment from gene expression data, we investigated if DRonA could be used to accelerate multicomponent drug discovery by predicting the outcome of drug interactions from single-drug-treated transcriptomes. To do this, we developed an approach to infer the transcriptomes of multidrug treatments. Specifically, we inferred the transcriptome of multidrug combinations by triangulation (see STAR Methods for details) of the respective transcriptomes obtained from single-drug-treated cultures of Mtb and then used the inferred multidrug transcriptome with DRonA to predict the CVS of the multidrug combination (i.e., the “predicted CVS”). Transcriptomes used for prediction of drug interactions were from Mtb treated with single drugs in matched experimental conditions (7H9 medium and 72 h drug treatment). Using this method to predict the CVS of multidrug combinations, we developed a parametric method, “MLSynergy”, to predict the interaction outcome of the two- and three-drug combinations. MLSynergy predicts the synergy or antagonism of multidrug combinations based on the Loewe additivity principle (Loewe, 1928) by calculating the ratio of predicted CVS to expected CVS, where the “expected CVS” for a drug combination is the average of CVSs from respective single-drug treatments (Figure 1). For example, the predicted CVS of the antagonistic combination linezolid and moxifloxacin (Cokol et al., 2017; Deshpande et al., 2016) is greater than the expected CVS and lies above the additive plane (Figure 5A), whereas the predicted CVS of the synergistic combination linezolid and POA is less than the expected CVS and lies below the additive plane (Figure 5B). Finally, the predicted CVS of linezolid with itself is the same as the expected CVS and lies on the additive plane, consistent with the Loewe additivity principle, which states that a drug in combination with itself is additive in interaction (Loewe, 1928) (Figure 5C). As such, an MLSynergy score <1 predicts that the drug interaction is synergistic, and a score >1 indicates an antagonistic drug interaction. We calculated MLSynergy scores for all two- and three-drug combinations of eight frontline drugs (Table S2). We compared MLSynergy predictions of 26 two-drug and 40 three-drug combinations of the eight frontline drugs with their experimentally determined interaction, quantified by fractional inhibitory concentrations (FICs) (Larkins-Ford et al., 2021). This comparative analysis demonstrated that MLSynergy was >90% accurate in predicting synergistic and antagonistic effects of two- and three-drug combinations (Figure 5D and 4E). Moreover, MLSynergy scores were highly correlated with the FIC values (*ρ* = 0.61, p value < 0.001, Figure S3). Interestingly, three two-drug combinations (identified with red font in Figure 5D) were predicted as synergistic by MLSynergy, but were determined to be antagonistic by DiaMOND assay (Larkins-Ford et al., 2021). Notably, these combinations were previously determined to be synergistic by other studies (Cokol et al., 2017; Diacon et al., 2012; Maltempe et al., 2017), suggesting that the effect of their drug interaction could be highly dependent on the assay method and conditions. Finally, we checked the ability of MLSynergy to predict condition-dependent drug interactions in Mtb, using as input 22 transcriptomes of Mtb from untreated and drug-treated infected macrophages of J774A.1 lineage (Table S2) (Liu et al., 2016). We predicted drug interaction for two- and three-drug combinations of isoniazid, rifampicin, and pyrazinamide in both broth culture and macrophages and compared the MLSynergy predictions with their experimental FIC values (Larkins-Ford et al., 2021) (Table S5). MLSynergy predicted that all the drug combinations are synergistic in 7H9 media and turn antagonistic in macrophage. Similarly, the experimental results found that mostly all the drug combinations (with the exception of isoniazid + rifampicin) are synergistic in broth and antagonistic in macrophage. This demonstrates that MLSynergy is robust to the context in which a drug effect is measured, and it can predict condition-dependent drug interactions. The benefit of MLSynergy to perform condition-dependent predictions will become clearer upon further availability of drug-treated transcriptomes of Mtb from macrophages and other *in vivo* contexts.

## Discussion

Here, we report the first machine learning framework for drug response prediction in Mtb. DRonA enables efficient prediction of cell viability from transcriptomic signatures of perturbation, including drug treatment. Using DRonA estimates of cell viability from single-drug transcriptomic data, MLSynergy can then predict synergy and antagonism of antitubercular drug combinations. Our analysis using DRonA found a strong association between *in silico* estimates of cell viability following drug treatment and experimentally observed reduction in CFUs. Moreover, the loss of viability captured by DRonA from Mtb transcriptomes of patients undergoing HRZE treatment supports the clinical utility of our approach. Finally, we report several synergistic drug combinations, suggesting that the DRonA/MLSynergy framework is a promising tool for the prioritization of new multicomponent drug regimens. While we validated predictions of two- and three-drug interactions, our framework is generalizable for higher-order combinations.

The suitability of using the transcriptome as a reflection of Mtb viability was studied by treating Mtb with seven frontline drugs and isolating RNA for transcriptome profiling, while also evaluating cell viability by CFU. The DRonA predicted the CVS of Mtb exposed to bactericidal (i.e., >MIC_50_) concentrations of drugs were below the cell viability threshold, proportional to relative CFU and significantly different from the CVS of untreated Mtb cultured for the same duration as drug treatment. Moreover, DRonA was able to perform effectively with other transcriptomic datasets of Mtb drug treatment, including during macrophage infection and from TB patients. The ability of DRonA to accurately predict the consequence of drug treatment in 7H9 medium, within macrophages, and from patient sputum, demonstrates that the definitions of viability in the DRonA model are inclusive of both actively dividing and slow replicating (physiologically adapted) phenotypes of Mtb. Moreover, the accuracy across datasets offers DRonA as a generalizable tool for use across drug response screens and in studies where gene expression was analyzed, but Mtb viability was not measured.

Here, we showed that DRonA complements bacteriological assays in evaluating treatment response. The decline in CVS corresponded to the decline in the proportions of surviving bacilli upon drug treatment, as measured by the relative CFU counts. Since no culturing is required, DRonA can estimate drug effects much faster than conventional bacteriological assays. Additionally, the ability to enrich and amplify RNA should allow DRonA to be used with samples where bacterial cell numbers are low (Mothershed and Whitney, 2006). The high sensitivity and the autonomy from culturing makes DRonA especially promising to evaluate the efficacy of treatment regimens on dormant non-culturable Mtb that are associated with latent infection in humans.

Using DRonA-predicted viability scores, MLSynergy accurately predicted synergy and antagonism for two- and three-drug combinations. This performance compares with INDIGO-MTB (Ma et al., 2019), an existing strategy that quantifies synergistic and antagonistic drug regimens using transcriptomes of Mtb treated with individual drugs, but only with drugs with known drug-drug interactions. INDIGO-MTB requires known drug-drug interactions to learn patterns and identify combinations most likely to be synergistic. In contrast, the DRonA/MLSynergy platform is based on gene signatures of cell viability and does not require any input data related to drug combinations. Comparing the accuracy for drugs without prior drug interaction information, MLSynergy significantly outperforms INDIGO-MTB (p value > 0.05, Figure S4). As such, our models can be more easily applied (i.e., no re-training required) to predict drug interaction for new drugs and conditions.

The DRonA/MLSynergy platform does have some potential limitations. First, our approach to predict cell viability based on gene expression signatures does not reveal information about drug mechanism of action. As cell death can lead to transcriptomic changes unrelated to drug treatment, these cell death signatures could be a confounding factor to make inferences about drug mechanism of action. Removing genes highly correlated to cell death could improve mechanism of action identification (Szalai et al., 2019). Future work also aims to integrate DRonA with regulatory-metabolic networks (Chandrasekaran and Price, 2010; Immanuel et al., 2021; Peterson et al., 2014; Turkarslan et al., 2015) to reveal the underlying pathways that contribute to a drug’s bactericidal activity and interaction with other drugs.

Second, the DRonA/MLSynergy platform requires transcriptome profiling of Mtb drug treatment to predict drug interactions. However, we argue that predicting drug interactions using transcriptome analysis with DRonA/MLSynergy is cheaper and faster, as compared with bacteriological assays. Evaluating drug interactions with bacteriological assays requires a significantly larger number of experiments, which increases exponentially with every new drug and for testing higher-order (i.e., three-drug) interactions. For example, to evaluate all possible two-drug interactions between 10 drugs (i.e., 45 combinations), a checkerboard or DiaMOND assay would require a minimum of 55 experiments, whereas MLSynergy would require just 10 experiments to generate transcriptomes of Mtb in response to treatment with each of the 10 drugs. For three-drug combinations, checkerboard or DiaMOND assay requirement increases to 120 drug dose titration experiments, whereas requirements for MLSynergy remains the same (i.e., 10 experiments). Furthermore, technological advancements are making it faster and cheaper to profile the transcriptome of Mtb directly from patient samples (Peterson et al., 2019), which could potentially extend the utility of DRonA in rapid point-of-care devices for evaluating the effectiveness of drug treatment in TB patients.

Drug response prediction with machine learning models is an important area of current research, particularly for a slow-growing pathogen, and our results highlight the practicality of using transcriptome signatures to address major bottlenecks in the drug discovery process. The ability to detect changes in cell viability and predict drug interaction using just transcriptome profiles could substantially accelerate TB drug discovery efforts. Recent studies have demonstrated that efficacy of the same drug combination can vary significantly between broth conditions and animal models (Larkins-Ford et al., 2021). DRonA and MLSynergy could be valuable for prioritizing drug combinations that are likely to be effective in animal models, given the challenges in performing high-throughput drug assays in mouse models and non-human primates. Finally, the DronA/MLSynergy framework can be easily extended to predict other genotypes and phenotypes of Mtb associated with a gain in drug resistance (e.g., metabolic states and cell wall composition), which could further improve treatment response prediction and clinical outcomes. To facilitate the widespread usage of these resources, the compendia of Mtb transcriptomes collated from GEO and generated in this study are publicly available, together with DRonA and MLSynergy algorithms and models in the MTB Network Portal (http://networks.systemsbiology.net/mtb/).

### Limitations of the study

While we have demonstrated that DRonA is effective in quantifying loss of viability of sensitive and resistant strains of Mtb in patient sputa, and even in mixed cultures consisting of dormant bacteria, we are yet to demonstrate the utility of this technique in quantifying persister load. Persisters of Mtb can survive drug pressure and cause treatment failure, and therefore, detecting and quantifying persisters is essential to ascertain complete bacterial clearance. Time-kill curves with CFU counting has been the gold standard for quantifying persisters at single-cell resolution. In principle, as and when technologies to profile Mtb transcriptomes at single-cell resolution are sufficiently mature, DRonA could replace CFU counting for accurate quantification of persisters from host-relevant conditions, including granulomas. Similarly, the DRonA/MLSynergy platform can predict whether drugs would combine synergistically or antagonistically in any given context from which Mtb transcriptomes can be derived. This capability is important, because the nature of drug interactions can vary dramatically from broth to *in vivo* conditions. The ability of generating sufficient numbers of transcriptome profiles of drug-treated Mtb from an *in vivo* context, such as from granuloma or from caseum, remains a key limitation in demonstrating the utility of DRonA and MLSynergy capability to predict drug interactions in an *in vivo* context. We could address this limitation by leveraging methods such as Path-seq to enrich and quantify Mtb transcriptomes from *in vivo* contexts.

## STAR★Methods

### Key resources table

**Table:** 

REAGENT or RESOURCE	SOURCE	IDENTIFIER
**Chemicals, peptides, and recombinant proteins**		

Middlebrook 7H9 Broth Base (7H9 broth)	Millipore-Sigma	M0178-500G
Middlebrook 7H10 Agar Base (7H10 agar)	Millipore-Sigma	M0303
Bedaquiline	Millipore-Sigma	843663-66-1
Clofazimine	Millipore-Sigma	2030-63-9
Isoniazid	Millipore-Sigma	54-85-3
Linezolid	Millipore-Sigma	165800-03-3
Moxifloxacin hydrocholoride	Millipore-Sigma	186826-86-8
Pretomanid	Millipore-Sigma	187235-37-6
Pyrazinecarboxylic acid (Pyrazinoic acid)	Millipore-Sigma	98-97-5
Rifampicin	Millipore-Sigma	13292-46-1
SuperScript II Reverse Transcriptase	ThermoFisher	18064014

**Critical commercial assays**		

Ribo-Zero Bacteria rRNA Removal Kit	Illumina	20040526
TruSeq Stranded mRNA HT library preparation kit	Illumina	20020595

**Deposited data**		

Transcriptomes from single drug treated Mtb	This paper	GEO: GSE165673
Mtb Transcriptome compendium for training of DRonA	This paper	GitHub: baliga-lab/DRonA_MLSynergy
Trained DRonA model (MTB_2020) used in this paper	This paper	GitHub: baliga-lab/DRonA_MLSynergy

**Experimental models: Organisms/strains**		

*Mycobacterium tuberculosis:* H37Rv	ATCC	27294

**Software and algorithms**		

Python	https://www.python.org/	N/A
SciPy	https://www.scipy.org/	N/A
GEOparser	This paper	Zenodo: https://doi.org/10.5281/zenodo.5598251, GitHub: baliga-lab/DRonA_MLSynergy
DRonA	This paper	Zenodo: https://doi.org/10.5281/zenodo.5598251, GitHub: baliga-lab/DRonA_MLSynergy
MLSynergy	This paper	Zenodo: https://doi.org/10.5281/zenodo.5598251, GitHub: baliga-lab/DRonA_MLSynergy
Google Colab notebook for DRonA and MLSynergy	This paper	Zenodo: https://doi.org/10.5281/zenodo.5598725, GitHub: baliga-lab/Google-colab-notebooks/blob/master/DRonA_MLSYnergy.ipynb

### Resource availability

#### Lead contact

Additional information and requests for resources and reagents should be directed to and will be fulfilled by the Lead Contact, Nitin S. Baliga (nitin.baliga@isbscience.org)

#### Materials availability

This study did not generate new unique materials nor reagents

### Experimental model and subject details

#### Bacterial strains and growth conditions

*Mycobacterium tuberculosis* strains used in this study was H37Rv. Mtb cells were culture in standard 7H9-rich media consisting of 7H9 broth with 0.05% Tween-80, 0.2% glycerol, and 10% Middlebrook ADC. Frozen 1 mL stocks of Mtb cells were added to 7H9 medium and grown with mild agitation in a 37°C incubator until the culture reached an OD_600_ of ∼0.4–0.8. The cells were then diluted to OD_600_ of 0.05 and added to 7H9-rich medium containing drugs at the predetermined amounts.

### Method details

#### Minimum inhibitory concentration 50 (MIC_50_) determination

10 mM working concentrations of drugs considered in the study were made with a suitable vehicle depending on drug solubility (*i.e.*, water, DMSO, or methanol). The 10 mM working concentrations of drugs were diluted in a twofold dilution series for 11 concentrations in 96-well plates. Each treatment series contained an untreated well as a control. Mtb H37Rv cultures were added to the wells and the plates were incubated at 37°C. Growth in cultures were measured as OD_600_ at 0 and 72 hours of incubation. All MIC_50_ determinations were performed in biological triplicate. Growth inhibition was determined by subtracting the initial reads from the final reads and then normalizing the data to no drug controls. Growth inhibition was fit to a sigmoidal curve and MIC_50_ was calculated for each drug (Table S3).

#### Time-kill assays

Using growth conditions described above, cells were diluted into 7H9-rich media containing drugs at predetermined amounts, along with vehicle controls (Table S3). Samples were taken after 0, 24 and 72 h, serially diluted and plated on 7H10 agar plates. All time-kill assays were performed in biological triplicate. Relative CFUs were calculated as log_10_ ratio of CFUs/ml of culture observed at start of treatment (T_0_) and after drug treatment.

#### Collection, RNA extraction, and analysis of single-drug transcriptomes

Using growth conditions described above, cells were diluted into 7H9-rich media containing drugs at predetermined amounts, along with vehicle controls (Tables S2 and S3). Samples, in biological triplicates, were collected after 24 and 72 h. Samples were centrifuged at high speed for 5 min, supernatant was discarded and cell pellet was immediately flash frozen in liquid nitrogen. Cell pellets were stored at −80°C until bead beating in a FastPrep 120 homogenizer and RNA extraction was performed as previously described (Peterson et al., 2020). Total RNA was depleted of ribosomal RNA using the Ribo-Zero Bacteria rRNA Removal Kit (Illumina, San Diego, CA). Quality and purity of the mRNA was determined with 2100 Bioanalyzer (Agilent, Santa Clara, CA). Sequencing libraries were prepared with TrueSeq Stranded mRNA HT library preparation kit (Illumina, San Diego, CA). All samples were sequenced on the NextSeq sequencing instrument in a high output 150 v2 flow cell. Paired-end 75 bp reads were checked for technical artifacts using Illumina default quality filtering steps. Raw FASTQ read data were processed using the R package DuffyNGS as previously described (Vignali et al., 2011). Read counts were further analyzed with Kallisto (Bray et al., 2016) and RPKM values were calculated.

#### Collection and curation of Mtb-GEO dataset for training of DRonA

GEOParser was developed to download transcript profiles and metadata of drug-treated and untreated samples of Mtb-H37Rv from Gene Expression Omnibus (GEO). GEOparser collected median spot intensity from microarray samples and Reads Per Kilobase of transcript, per Million mapped reads (RPKM) from RNA-seq samples. The compendium dataset was curated by removing samples with low coverage (i.e., samples with <70% of annotated Mtb genes). The curated dataset was normalized by rank normalization with the “minimum” method with SciPy package (Virtanen et al., 2020).

#### Manual labeling of Mtb transcriptomes

Using the metadata collected by GEOParser (Table S1), transcriptomes were labelled as “viable” if the sample description stated that Mtb cultures were grown in optimal growth conditions (mid-log phase of growth in 7H9-rich media, incubated at 37°C with aeration) and “non-viable” if the sample description stated that Mtb cultures were treated with >1x MIC_50_ drug for >12 hours. The remaining transcriptomes were labeled as “unclassified”. Labels were saved as a comma separated value (.csv) file (Table S1).

#### Training and running DRonA

Rank, normalized transcriptomes along with the labels (Table S1) was provided to the SC-SVM classifier to start the iterative training of DRonA. Each iteration consisted of the following steps: (1) a single class support vector machine (SC-SVM) was trained on the training set i.e. transcriptomes labelled as “viable”; (2) the accuracy of the trained SC-SVM was calculated with Equation 1 using the test set i.e. transcriptomes labelled as “non-viable” initially and ones classified as “viable” through the iteration process;

(Equation 1) $$Accuracy=\frac{True\;positive+True\;negatives}{True\;positive+True\;negatives+False\;positive+False\;negative}$$

(3) assessment of the accuracy, (4) using the trained SC-SVM from (1), viability was predicted in transcriptomes labelled as “unclassified”, and (5) newly predicted viable transcriptomes from unclassified set were moved to the training set. The iterative process was stopped when the accuracy of the classifier dropped below an accuracy threshold (85%) or when no new transcriptomes from the unclassified set were found to be viable. The cell viability scores (CVS) were calculated for samples as the weighted sum of gene expression ranks using the trained SC-SVM. CVSs were normalized by subtracting the score of a sample with maximum score observed in that experiment.

#### Inference of multi-drug transcriptomes (triangulation)

Transcriptomes of the Mtb cultures treated with multi-drug combinations at effective doses were predicted by triangulation with the single-drug treated transcriptomes and untreated control. Triangulation was called through ‘triangulate’ function in the MLSynergy algorithm, which collects transcriptomes of the drugs in combination (each profiled as single-drug) and untreated control and averages them with geometric mean. The inferred multi-drug transcriptomes were then returned to DRonA for CVS determination.

#### Calculation of MLSynergy scores for drug combinations

Expected CVSs were obtained from DRonA with the transcriptomes of the single-drug treatments that make up the drug combination and “expected CVS” was calculated by averaging the CVSs of single-drug treatments. The “predicted CVS” was obtained from DRonA with the inferred transcriptome of the drug combination. MLSynergy score were calculated as the ratio of expected CVS and predicted CVS. Further, MLSynergy scores were log normalized (base 2) in reference to the average of MLSynergy scores of same drug combinations that are considered to be additive in nature.

#### Comparison of INDIGO-MTB and MLSynergy predictions

Two INDIGO (Ma et al., 2019) models were retrained with default parameters. Model-1 was trained with the complete dataset (202 combinations and 46 drugs) and Model-2 was trained with partial dataset (98 combinations and 40 drugs) which was obtained after excluding combinations with bedaquiline, clofazimine, linezolid, moxifloxacin, pretomanid and pyrazinamide. Both models were tested on the combinations given in Table S6. Transcriptomes provided in Ma et al. were used as input for the INDIGO models. Transcriptomes generated in this study (summarized in Table S2) were used as input for the MLSynergy.

### Quantification and statistical analysis

All statistical analysis reported in this article were performed with SciPy package (Virtanen et al., 2020) in Python. The p value from the Student’s t test, sample mean and SEM were used as indicated in Figures 2, 3, 4, 5D, S3, and S4. Statistically non-significant (NS) were considered with *p* value > 0.05 and other qualifying *p* values were indicated accordingly ∗ < 0.05, ∗∗ < 0.01, and ∗∗∗< 0.001. The correlations reported in Figures 3 and S3 were calculated as the average correlation coefficient, r, from 100 iterations performed with 70% randomly selected data, *r* and *p* values were reported in the figures.

## Data Availability

• The raw sequencing data have been deposited in GEO with accession number GSE165673. Information also listed in the key resources table. • All original code for GEOparser, DRonA and MLSynergy has been deposited in Zenodo at https://doi.org/10.5281/zenodo.5598251, GitHub (baliga-lab/DRonA_MLSynergy), and MTB Network Portal (http://networks.systemsbiology.net/mtb/). Implementation of DRonA and MLSynergy on Google Colab server has been deposited in Zenodo at https://doi.org/10.5281/zenodo.5598725 and GitHub (baliga-lab/Google-colab-notebooks/blob/master/DRonA_MLSYnergy.ipynb). Information also listed in the key resources table. • Any additional information required to analyze the data reported in this paper is available from the lead contact upon request.

## Supplemental information

Document S1. Figures S1–S4 and Tables S2–S6

Table S1. Metadata of the GEO compendium used for training DRonA, related to manual labelling of Mtb transcriptomes and training and running DRonA sections in STAR Methods

Document S2. Article plus supplemental information
