# Supplementary material for: Transcriptome signature of cell viability predicts drug response and drug interaction in Mycobacterium tuberculosis
Source: Cell Rep Methods. 2021 Dec 6;1(8):100123. doi: 10.1016/j.crmeth.2021.100123 (PMC8688151; doi:10.1016/j.crmeth.2021.100123)
Supplement: Document S1. Figures S1–S4 and Tables S2–S6 [file mmc1.pdf]

Cell Reports Methods, Volume *1*

Supplemental information

Transcriptome signature of cell viability predicts drug response and drug interaction in *Mycobacterium tuberculosis*

Vivek Srinivas, Rene A. Ruiz, Min Pan, Selva Rupa Christinal Immanuel, Eliza J.R. Peterson, and Nitin S. Baliga

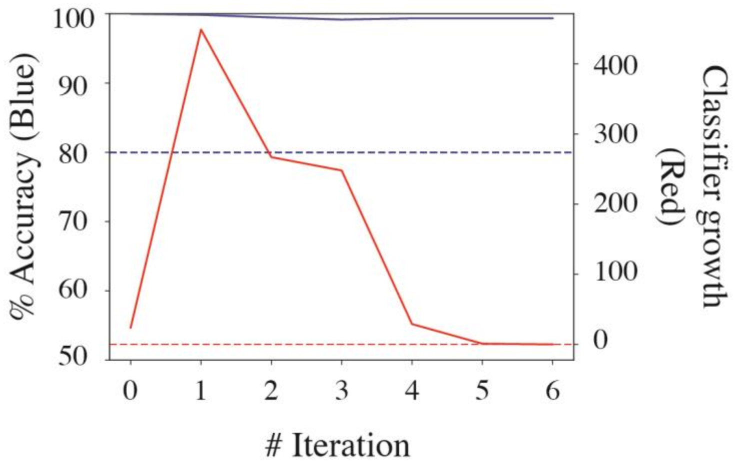

Figure S1.

Iterative training of DRonA, related to training and running DRonA section in STAR methods. The graph shows changes in accuracy and number of unclassified transcriptomes added to the viable training (Classifier growth in Red) at each iteration. The % accuracy was calculated with Eq.1 with viable and non-viable set of transcriptomes. Blue and red dashed line show the threshold for % accuracy and classifier growth below which the iterative training was programmed to stop. The classifier growth dropped below threshold at the fifth iteration and was halted.

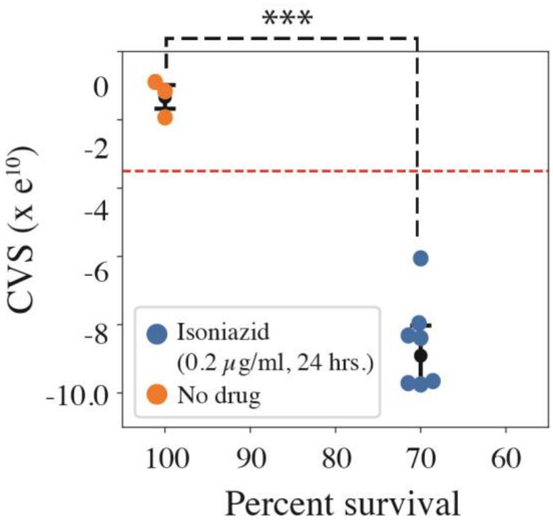

Figure S2.

CVS accurately predicts bactericidal effects of INH on Mtb in intracellular environment, related to Figure 3. CVS was calculated using DRonA analysis of Mtb transcriptomes from infected macrophages. CVS was correlated with percent survival generated from CFU data for intracellular Mtb with and without 0.2 µg/ml isoniazid (INH) treatment (*Liu et al., 2016*). The dashed red line is the cell viability threshold ($-3.5e^{10}$), below which the samples are considered to be non-viable. Black dot and error bars indicate the mean and standard deviation away from the mean. Statistical significance (black dashed line) was calculated as *p*-value with Student's T-test. ***: p-value < 0.001.

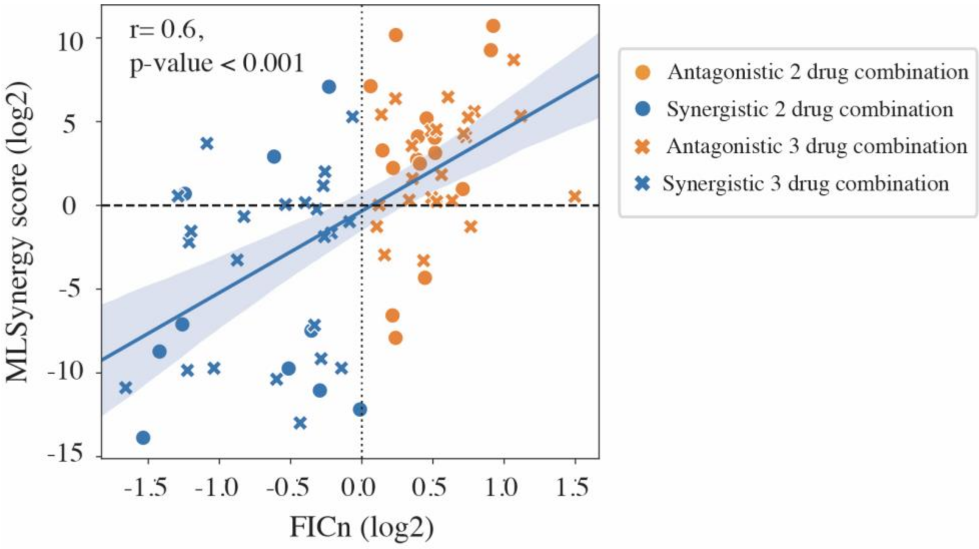

Figure S3.

Correlation between MLSynergy score and FICs *(Cokol et al., 2017; Larkins-Ford et al., 2021)* for two- and three-drug combinations, related to Figure 5D. Solid blue line denotes the Pearson's correlation between CVS and relative CFU. Significance was calculated as the average correlation coefficient, *r*, from 100 iterations performed with 70% randomly selected data. Black dotted line and black dashed line are the FICs and MLSynergy scores, respectively, that separate synergistic combinations from the antagonistic combinations.

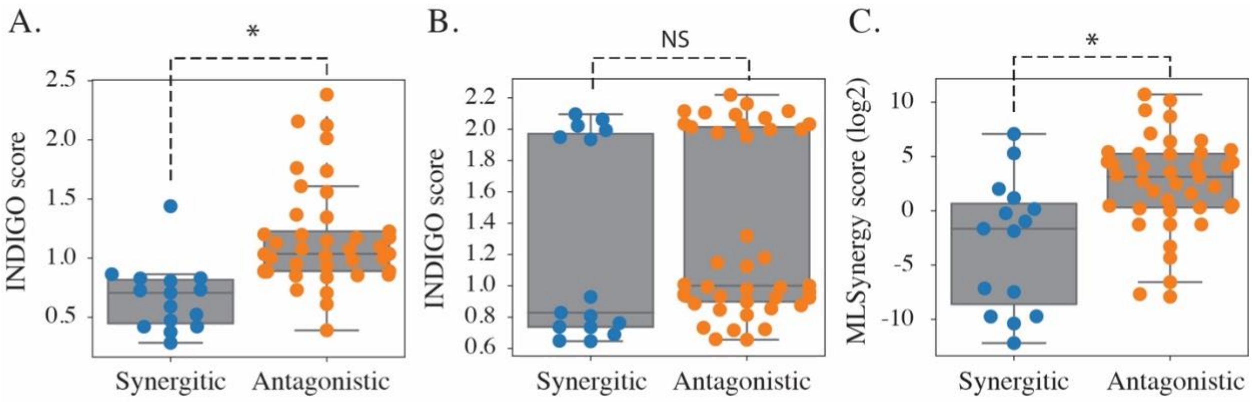

Figure S4.

Comparison of INDIGO *(Ma et al., 2019)* models with MLSynergy in predicting interaction of 2- and 3-drug combinations, related to Figure 5D. (**A**) Predictions from INDIGO model that was trained on 202 drug combinations with 46 drugs (Model-1) (**B**) Predictions from INDIGO model that was trained on 98 drug combination with 40 drugs (Model-2); specifically combinations with bedaquiline, clofazimine, linezolid, moxifloxacin, pretomanid and pyrazinamide were excluded from training. (**C**) Predictions from MLSynergy model with same drug combinations as (B). Drugs were validated as synergistic and antagonistic from DiaMOND assay. Statistical significance (black dashed line) was calculated as *p*-value with Student's T-test. *: p-value < 0.05. Data plotted is tabulated in Table S7.

Drug	Concentration (μg/ml)	Treatment time (hrs.)	Growth context	Replicates	Study
BDQ	11.5	72	Broth	3	This study
CFZ	3.65	72	Broth	3	This study
INH	1.8	72	Broth	3	This study
LZD	4.2	72	Broth	3	This study
MXF	0.3	72	Broth	3	This study
PA824	0.7	72	Broth	3	This study
POA	3.5	72	Broth	3	This study
RIF	0.02	72	Broth	3	This study
No drug (Lag phase)	0	0	Broth	12	This study
No drug (Early log phase)	0	0	Broth	12	This study
No drug (Log phase)	0	0	Broth	12	This study
No drug (Stationary phase)	0	0	Broth	12	This study
EMB	12	24	Broth	3	*Liu et al., 2016*
INH	0.4	24	Broth	6	*Liu et al., 2016*
POA	200	24	Broth	3	*Liu et al., 2016*
RIF	0.4	24	Broth	2	*Liu et al., 2016*
EMB	12	24	Intra-macrophage	3	*Liu et al., 2016*
INH	0.4	24	Intra-macrophage	8	*Liu et al., 2016*
POA	200	24	Intra-macrophage	3	*Liu et al., 2016*
RIF	0.4	24	Intra-macrophage	3	*Liu et al., 2016*
No drug (Inf-g)	0	24	Intra-macrophage	2	*Liu et al., 2016*
No drug (PBS)	0	24	Intra-macrophage	4	*Liu et al., 2016*
No drug (RAP)	0	24	Intra-macrophage	2	*Liu et al., 2016*

Table S2.

Treatments used to generate the transcriptomes used to test DRonA and predict drug interactions with MLSynergy, related to Figure 2 - 5.

Drug	Low concentration (μg/ml)	High concentration (μg/ml)	MIC50 (μg/ml)	Related to Figure 3 (number legend)
Bedaquiline	5.75	11.5	1.61	(1) 5.75 μg/ml, 24 h<br>(2) 5.75 μg/ml, 72 h
Clofazimine	0.73	3.65	1.17	(3) 0.73 μg/ml, 24 h<br>(4) 0.73 μg/ml, 72 h
Isoniazid	0.36	1.8	0.2	(5) 0.36 μg/ml, 24 h<br>(6) 0.36 μg/ml, 72 h<br>(7) 1.8 μg/ml, 24 h<br>(8) 1.8 μg/ml, 72 h
Linezolid	0.84	4.2	1.13	(9) 0.84 μg/ml, 24 h<br>(10) 0.84 μg/ml, 72 h
Moxifloxacin	0.12	0.3	0.07	(11) 0.12 μg/ml, 24 h<br>(12) 0.12 μg/ml, 72 h<br>(13) 0.3 μg/ml, 24 h<br>(14) 0.3 μg/ml, 72 h
No drug	NA	NA	NA	(15) NA, 24 h<br>(16) NA, 72 h
Pretomanid	0.7	3.5	0.15	(17) 0.70 μg/ml, 24 h<br>(18) 0.70 μg/ml, 72 h
Rifampicin	0.008	0.02	0.02	(19) 0.008 μg/ml, 24 h<br>(20) 0.008 μg/ml, 72 h<br>(21) 0.02 μg/ml, 24 h<br>(22) 0.02 μg/ml, 72 h

Table S3.

Drug concentrations used for transcriptome profiles generated in this study. The low (bacteriostatic) and high (bactericidal) drug concentrations were selected based on time-kill assays. The MIC50 determination for drugs used in this study, related to Minimum Inhibitory Concentration 50 (MIC50) determination section in STAR methods. The number legend for Figure 3, drug treatment concentrations and time used to compare the DRonA generated CVS and relative CFUs.

Sample #	Drug	Concentration (μg/ml)	CFUs /ml (10^6)			Relative CFUs (log10)	
			h0	h24	h72	h24	h72
2	BDQ	1.15	61	91	352	0.174	0.761
3	BDQ	1.15	69	96	362	0.143	0.72
4	BDQ	1.15	72	101	346	0.147	0.682
5	BDQ	5.75	55	97	115	0.246	0.32
6	BDQ	5.75	53	81	132	0.184	0.396
7	BDQ	5.75	61	100	96	0.215	0.197
18	CFZ	0.0728	148	124	207	-0.077	0.146
19	CFZ	0.0728	239	116	176	-0.314	-0.133
20	CFZ	0.0728	141	112	192	-0.1	0.134
21	CFZ	0.728	158	87	41	-0.259	-0.586
22	CFZ	0.728	182	69	26	-0.421	-0.845
23	CFZ	0.728	101	67	52	-0.178	-0.288
36	INH	0.018	49	182	184	0.57	0.575
37	INH	0.018	91	160	194	0.245	0.329
38	INH	0.018	132	171	203	0.112	0.187
39	INH	0.18	93	60	78	-0.19	-0.076
40	INH	0.18	77	90	97	0.068	0.1
41	INH	0.18	70	60	70	-0.067	0
42	INH	0.36	159	1	0.1	-2.201	-3.201
43	INH	0.36	180	4	0.1	-1.653	-3.255
44	INH	0.36	187	3	0.1	-1.795	-3.272
45	INH	1.8	77	1	0.1	-1.886	-2.886
46	INH	1.8	68	3	0.1	-1.355	-2.833
47	INH	1.8	87	4	0.1	-1.337	-2.94
63	LZD	0.0844	81	135	249	0.222	0.488
64	LZD	0.0844	100	147	245	0.167	0.389
65	LZD	0.0844	98	127	239	0.113	0.387
66	LZD	0.844	61	63	71	0.014	0.066
67	LZD	0.844	68	74	86	0.037	0.102
68	LZD	0.844	60	92	72	0.186	0.079
78	MXF	0.075	69	90	159	0.115	0.363
79	MXF	0.075	53	88	165	0.22	0.493
80	MXF	0.075	72	80	147	0.046	0.31
81	MXF	0.3	43	4	0.1	-1.031	-2.633
82	MXF	0.3	50	9	0.1	-0.745	-2.699

83	MXF	0.3	49	7	0.1	-0.845	-2.69
1	No drug	0	48	178	556	0.569	1.064
15	No drug	0	68	148	376	0.338	0.743
16	No drug	0	116	168	235	0.161	0.307
17	No drug	0	154	178	197	0.063	0.107
33	No drug	0	165	164	231	-0.003	0.146
34	No drug	0	177	205	219	0.064	0.092
35	No drug	0	128	204	313	0.202	0.388
60	No drug	0	89	173	336	0.289	0.577
61	No drug	0	86	175	378	0.309	0.643
62	No drug	0	67	107	353	0.203	0.722
90	No drug	0	76	157	248	0.315	0.514
91	No drug	0	49	173	187	0.548	0.582
92	No drug	0	99	223	119	0.353	0.08
114	No drug	0	51	205	324	0.604	0.803
128	No drug	0	42	198	368	0.673	0.943
93	PA824	0.07	115	213	204	0.268	0.249
94	PA824	0.07	51	236	189	0.665	0.569
95	PA824	0.07	32	174	192	0.735	0.778
96	PA824	0.7	37	86	15	0.366	-0.392
97	PA824	0.7	78	84	20	0.032	-0.591
98	PA824	0.7	83	82	16	-0.005	-0.715
99	PA824	3.5	125	105	4	-0.076	-1.495
100	PA824	3.5	96	114	0	0.075	0
101	PA824	3.5	100		3	0	-1.523
115	RIF	0.005	60	121	179	0.305	0.475
116	RIF	0.005	50	105	152	0.322	0.483
117	RIF	0.005	49	134	137	0.437	0.447
118	RIF	0.02	71	43	10	-0.218	-0.851
119	RIF	0.02	50	54	14	0.033	-0.553
120	RIF	0.02	56	54	11	-0.016	-0.707

Table S4.

Relative CFUs from single drug treated time kill curves of Mtb cultures, related to Figure 3. Relative CFU was measured as a ratio between the CFUs observed at start of the treatment (h0) vs. CFUs observed post treatment.

Drug			MLSynergy scores		FIC_{50} value (log_2)	
1	2	3	7H9	Macrophage	7H9	Macrophage
PZA	RIF		-8.72	2.7	-1.42	-0.11
INH	PZA		-7.1	2.0	-1.26	0.42
INH	RIF		-4.3	2.8	0.44	-0.07
INH	PZA	RIF	-7.13	2.45	-5.81	0.29

Table S5.

MLSynergy scores and FIC_{50} values of two- and three-drug combinations in broth and macrophage context, related to Figure 5D. MLSynergy predicted and experimentally determined (*Larkins-Ford et al., 2021)* interactions of pyrazinamide (PZA), isoniazid (INH), and rifampicin (RIF) from Mtb growing in 7H9 media or infected J774A.1 macrophages. Drug combinations with MLSynergy score and FIC value (log_2) < 0 are considered synergistic and > 0 are considered antagonistic in interaction.

Drug 1	Drug 2	Drug 3	Interaction type (DiaMOND interpreted)	MLSynergy score	Interaction type (MLSynergy interpreted)	INDIGO score (Model 1)	INDIGO score (Model 2)
BDQ	CFZ		Synergy	7.08	Synergy	0.28	2.10
BDQ	INH		Antagony	7.12	Synergy	1.20	2.22
BDQ	LZD		Antagony	10.17	Synergy	1.20	2.09
BDQ	MXF		Antagony	10.72	Synergy	2.38	2.16
BDQ	PA824		Antagony	2.74	Synergy	1.04	2.12
BDQ	POA		Synergy	2.91	Synergy		
BDQ	RIF		Antagony	4.11	Synergy	1.76	2.12
CFZ	INH		Antagony	-6.57	Synergy	0.39	0.95
CFZ	LZD		Antagony	0.98	Synergy	0.98	0.66
CFZ	MXF		Antagony	3.13	Synergy	1.74	0.85
CFZ	PA824		Synergy	-12.18	Synergy	1.44	0.83
CFZ	POA		Synergy	-11.05	Synergy		
CFZ	RIF		Synergy	-7.47	Synergy	0.47	0.65
INH	LZD		Antagony	3.28	Synergy	0.86	0.99
INH	MXF		Antagony	5.19	Synergy	2.01	1.18
INH	PA824		Antagony	-7.91	Synergy	1.00	1.13
INH	POA		Synergy	-7.10	Synergy		
INH	RIF		Antagony	-4.32	Synergy	1.37	1.32
LZD	MXF		Antagony	9.27	Synergy	1.56	1.00
LZD	PA824		Antagony	0.35	Synergy	1.61	0.96
LZD	POA		Synergy	0.69	Synergy		
LZD	RIF		Antagony	2.23	Synergy	0.95	0.72
MXF	PA824		Antagony	2.49	Synergy	2.16	1.15
MXF	RIF		Antagony	4.04	Synergy	2.12	0.99
PA824	POA		Synergy	-13.87	Synergy		
PA824	RIF		Synergy	-9.74	Synergy	0.42	0.93
POA	RIF		Synergy	-8.72	Synergy		
BDQ	CFZ	INH	Synergy	2.01	Synergy	0.38	1.99
BDQ	CFZ	LZD	Synergy	5.29	Synergy	0.81	1.94
BDQ	CFZ	MXF	Antagony	6.38	Synergy	0.89	2.00
BDQ	CFZ	PA824	Synergy	-0.22	Synergy	0.86	2.02
BDQ	CFZ	POA	Synergy	0.05	Synergy		
BDQ	CFZ	RIF	Synergy	1.17	Synergy	0.42	1.95
BDQ	INH	LZD	Antagony	5.42	Synergy	0.85	1.95

BDQ	INH	MXF	Antagony	6.47	Synergy	1.17	2.07
BDQ	INH	PA824	Antagony	0.31	Synergy	0.71	2.03
BDQ	INH	POA	Synergy	0.56	Synergy		
BDQ	INH	RIF	Antagony	1.60	Synergy	1.23	2.03
BDQ	LZD	MXF	Antagony	8.69	Synergy	1.10	2.00
BDQ	LZD	PA824	Antagony	3.56	Synergy	1.00	2.01
BDQ	LZD	POA	Synergy	3.70	Synergy		
BDQ	LZD	RIF	Antagony	4.47	Synergy	0.89	1.98
BDQ	MXF	PA824	Antagony	4.53	Synergy	1.04	2.11
BDQ	MXF	RIF	Antagony	5.34	Synergy	1.35	2.03
BDQ	PA824	POA	Synergy	-2.19	Synergy		
BDQ	PA824	RIF	Synergy	-0.97	Synergy	0.59	2.06
BDQ	POA	RIF	Synergy	-0.66	Synergy		
CFZ	INH	LZD	Antagony	-1.27	Synergy	0.73	0.72
CFZ	INH	MXF	Antagony	0.47	Synergy	0.89	0.88
CFZ	INH	PA824	Synergy	-9.71	Synergy	0.73	0.74
CFZ	INH	POA	Synergy	-9.15	Synergy		
CFZ	INH	RIF	Synergy	-7.15	Synergy	0.52	0.76
CFZ	LZD	MXF	Antagony	4.10	Synergy	1.02	0.73
CFZ	LZD	PA824	Antagony	-3.30	Synergy	1.08	0.66
CFZ	LZD	POA	Antagony	-2.96	Synergy		
CFZ	LZD	RIF	Synergy	-1.64	Synergy	0.71	0.65
CFZ	MXF	PA824	Antagony	-1.26	Synergy	1.13	0.81
CFZ	MXF	RIF	Synergy	0.16	Synergy	0.83	0.74
CFZ	PA824	POA	Synergy	-12.99	Synergy		
CFZ	PA824	RIF	Synergy	-10.38	Synergy	0.83	0.69
CFZ	POA	RIF	Synergy	-9.72	Synergy		
INH	LZD	MXF	Antagony	5.60	Synergy	1.14	0.94
INH	LZD	PA824	Antagony	-1.26	Synergy	0.89	0.92
INH	LZD	POA	Synergy	-0.96	Synergy		
INH	LZD	RIF	Antagony	0.22	Synergy	0.84	0.90
INH	MXF	PA824	Antagony	0.54	Synergy	1.03	0.98
INH	MXF	RIF	Antagony	1.83	Synergy	1.17	1.01
INH	PA824	POA	Synergy	-9.85	Synergy		
INH	PA824	RIF	Antagony	-7.67	Synergy	0.61	0.93
INH	POA	RIF	Synergy	-7.14	Synergy		
LZD	MXF	PA824	Antagony	4.29	Synergy	1.08	0.92
LZD	MXF	RIF	Antagony	5.25	Synergy	0.93	0.86
LZD	PA824	POA	Synergy	-3.26	Synergy		
LZD	PA824	RIF	Synergy	-1.87	Synergy	0.73	0.81

LZD	POA	RIF	Synergy	-1.54	Synergy		
MXF	PA824	RIF	Antagony	0.02	Synergy	0.85	0.89
MXF	POA	RIF	Antagony	0.29	Synergy		
PA824	POA	RIF	Synergy	-10.88	Synergy		

Table S6.

MLSynergy and INDIGO scores for 2- and 3-drug combinations, related to Figure 5D and S4.

Title and legend for Document S2 with Excel tables - Table S1

Table S1. Metadata of the GEO compendium used for training DRonA. Related to Manual labelling of Mtb transcriptomes and Training and running DRonA sections in STAR methods.
